# The Occurrence and Biological Activity of Tormentic Acid—A Review

**DOI:** 10.3390/molecules26133797

**Published:** 2021-06-22

**Authors:** Marta Olech, Wojciech Ziemichód, Natalia Nowacka-Jechalke

**Affiliations:** Chair and Department of Pharmaceutical Botany, Medical University of Lublin, 1 Chodźki Street, 20-093 Lublin, Poland; zimiwoj@gmail.com (W.Z.); natalia.nowacka@umlub.pl (N.N.-J.)

**Keywords:** tormentic acid, triterpenes, pentacyclic triterpene, CAS 13850-16-3, bioactivity, plant metabolite, tormentic acid derivatives

## Abstract

This review focuses on the natural sources and pharmacological activity of tormentic acid (TA; 2α,3β,19α-trihydroxyurs-2-en-28-oic acid). The current knowledge of its occurrence in various plant species and families is summarized. Biological activity (e.g., anti-inflammatory, antidiabetic, antihyperlipidemic, hepatoprotective, cardioprotective, neuroprotective, anti-cancer, anti-osteoarthritic, antinociceptive, antioxidative, anti-melanogenic, cytotoxic, antimicrobial, and antiparasitic) confirmed in in vitro and in vivo studies is compiled and described. Biochemical mechanisms affected by TA are indicated. Moreover, issues related to the biotechnological methods of production, effective eluents, and TA derivatives are presented.

## 1. Introduction

Given the constantly growing number of diseases and the common phenomenon of drug resistance, scientists are forced to seek for more potent and less toxic treatments. Pentacyclic triterpenes represent a valuable group of compounds among natural metabolites. They are abundant in the plant kingdom and are found in various plant parts, including edibles (olive, strawberries, mango, rose fruits, apples, mulberry, quince), herbs, and herbal products. Therefore, the quantities of these compounds in human diet can be quite significant. The individual average human intake of triterpenes was determined to be approximately 250 mg per day in the Western world, and even 400 mg per day in the Mediterranean countries [1].

Pentacyclic triterpenes have been repeatedly proven to possess a broad spectrum of pharmacological activities. The health-beneficial properties of these compounds have been shown to include anti-inflammatory, anticancer, antidiabetic, cardio- and hepato-protective, antimicrobial, antiviral, antiparasitic, and other activities [2,3,4,5,6]. The affinity and spectrum of biological activity is associated with the diverse triterpene skeleton structure and connected substituents. Even structurally quite similar triterpenes may have different pharmacological potential, polarity, solubility, and bioavailability and can occur in unrelated plant species [1,7,8,9]. One of the pentacyclic triterpenes is 2α,3β,19α-trihydroxyurs-2-en-28-oic acid known as tormentic acid (TA).

There are numerous available data on the occurrence of tormentic acid in different organs of different plant species, the types of extraction used for obtaining this compound, and the wide spectrum of biological activity of TA and extracts containing TA. However, there is no comprehensive review collecting such data. Therefore, the presented review summarizes the knowledge of the confirmed botanical sources, pharmacological activity of tormentic acid, biochemical mechanisms that can be affected by this molecule, and its most common natural derivatives.

## 2. Structure, Function, and Occurrence of TA

Tormentic acid, also known as 2α,3β,19α-trihydroxyurs-2-en-28-oic acid (IUPAC Name: (1*R*,2*R*,4*aS*,6*aR*,6*aS*,6*bR*,8*aR*,10*R*,11*R*,12*aR*,14*bS*)-1,10,11-trihydroxy-1,2,6*a*,6*b*,9,9,12*a*-heptamethyl-2,3,4,5,6,6*a*,7,8,8*a*,10,11,12,13,14*b*-tetradecahydropicene-4*a*-carboxylic acid), is a compound classified as a pentacyclic triterpene. Triterpenes are synthesized via the mevalonic acid (MVA) pathway in cytosol by cyclization of the squalene molecule. Their skeleton is composed of six isoprene units (C_5_). Owing to the number of cyclic structures making up such compounds, there is a wide variety of triterpenes, including pentacyclic triterpenes. These can be further categorized into the oleanane, ursane, lupine, and hopane groups. Tormentic acid belongs to ursane-type pentacyclic triterpenes [10,11].

Pentacyclic triterpenes act as phytoalexins, which play a significant role in plant defense against numerous pathogens and pests [12]. Several pentacyclic triterpenes have been found to possess insect antifeedant and toxic effects. Betulin derivatives are effective against *Heliothis zea* and *Leptinotarsa decemlineata*, while betulinic acid derivatives have activity against *Spodoptera litura*. Oleanolic acid derivatives possess anti-insect properties against *L. decemlineata*. Ursolic acids and its derivatives inhibit the growth of *S. litura.* Ursane-type triterpenes are antifeedants to *S. littoralis* [13]. Tormentic acid was isolated from strawberry (*Fragaria ananassa* cv. Houkouwase) and identified as one of the major phytoalexins responsible for resistance to the fungus *Colletotrichum fragariae* causing strawberry anthracnose [14]. Therefore, its production is probably related to plant defense mechanisms. 

Tormentic acid has been found in various species and plant families (Table 1). Based on the collected data, it can be assumed that this compound is typical of the Rosaceae family. However, several species from Lamiaceae and Urticaceae were also reported to be sources of this compound. Moreover, TA was also found in nineteen other families including, e.g., Betulaceae, Boraginaceae, Compositae, Caryophyllaceae, Ericaceae, Oleaceae, Polygonaceae, Urticaceae, and Saxifragaceae. The presence of this triterpene was revealed in different aerial and underground plant organs [3,15,16,17,18,19,20,21,22,23,24,25]. 

As already mentioned, the majority of species that are a confirmed source of TA belong to Rosaceae. Most commonly, TA was isolated from leaves and whole herb of rose family members [26,27,28,29,30,31]. However, a few studies indicated this metabolite in roots, seeds, or fruits as well [32,33,34].

*Eriobotrya japonica* (Thunb.) Lindl leaf is the best-recognized and rich source of TA. The compound was efficiently extracted from this plant material with 95% ethanol [31,35] or aqueous methanol [36]. Using extraction at elevated temperature (90 °C) and 95% ethanol, TA was obtained from dried powder of loquat leaf with a 5.8% yield [31]. Several studies have also reported large amounts of triterpenes (including TA) in callus cultures induced from *E. japonica* axenic leaves reaching 20–50 mg/g dry weight [4,37,38,39]. As reported by Taniguchi et al. [38], the isolation of the triterpenoids from callus tissues was easier than from the intact plant, due to the absence of chlorophylls in the callus. Using ultrasonic extraction of cell suspension culture from immature embryos from *E. japonica*, Li et al., (2017) [39] isolated TA with a yield higher than from dried leaves (63.1 mg/g dry weight). A similar yield was detected by Ho et al. [40]. It was also reported that this triterpene can be easily separated and purified with the use of preparative HPLC, which was confirmed by UPLC-MS analysis [39]. Other *E. japonica* organs (stem, root) were used to induce TA-rich callus cultures [40]. However, the leaf-derived culture was found to be the most promising. 

In the Lamiaceae family, TA was found, e.g., in flowering herb of *Lavendula luisieri, Perilla frutescens* leaves, and underground and aerial parts of sage [41,42,43,44]. It was also observed in callus culture induced from *P. frutescens* leaves, where it was reported to be one of the leading triterpenoids constituting approximately 1 mg/g of dry weight [45]. Again, this metabolite was successfully extracted from plant material with the use of simple and cheap techniques (like extraction in a Soxhlet apparatus or maceration) and with the use of safe, non-toxic solvents like ethanol [41,42,45,46]. However, efficient elution can be performed with the use of other solvents as well [47].

Many species from the Urticaceae family were found as a source of TA as well. TA was isolated from their stems, bark, roots, and less often leaves or whole aerial parts. For example, TA was found to occur in *Myrianthus arboreus* stems and root wood. The extractions were conducted using methylated spirit and chloroform or methylated ethyl acetate for root wood and stems, respectively [48,49,50]. 

Biotechnological methods (e.g., cell suspension cultures) were found to be an effective tool for extraction of increased portions of TA from tested species [23,40,45]. It is not known, however, whether a similar effect would be achieved in cultures derived from other organisms. Moreover, to date, relatively fewer studies have addressed the optimization of conditions for increased production or elution of TA from plant material. Both issues seem to be interesting, e.g., from the point of view of possible industrial production.

## 3. Pharmacological Activity of TA

Tormentic acid was found to possess various biological activities, including anti-inflammatory [33], antidiabetic, hypoglycemic [4,79], hepato-, neuro-, cardio-protective [18,77,94], anticancer, cytotoxic, antiproliferative [58,85,95], anti-osteoarthritic [96], antinociceptive [92], antibacterial [51], antiviral [71], and insect antifeedant [41] activities. The molecule was investigated in both in vitro and in vivo assays. Table 2 summarizes available data on TA activities and mechanisms of its action.

### 3.1. Anti-Inflammatory Activity

The protective effect of TA against inflammation occurs through a decrease in the levels of various pro-inflammatory mediators. The anti-inflammatory activity of TA involves inhibition of the expression of cyclooxygenase-2 (COX-2) and production of prostaglandins, interleukins, and thromboxane. Moreover, TA inhibits the production of nitric oxide (NO), influences the level of cytokines, and inhibits the production of interleukins IL-1B, IL-6 [99], and IL-8 [99,101]. The use of TA decreases the production of tumor necrosis factor-α (TNF-α) and inhibits activation of the NF-κB pathway [97,98,99]. Furthermore, TA is able to decrease reactive oxygen species generation (which constitutes its antioxidative activity) and inhibit H_2_O_2_-induced expression of inducible nitric oxide synthase (iNOS) and NADPH oxidase (NOX) [99]. TA was found to inhibit LPS-induced inflammation by suppressing the mitogen-activated protein kinase (MAPK) signaling pathway [101]. The anti-inflammatory activity of the compound was confirmed by both in vitro and in vivo studies. The activity in vivo was demonstrated in carrageenan-induced edema in Sprague-Dawley rats. TA from *E. japonica* cell suspension administered in a dose of 2.5 mg/kg inhibited paw edema after 4 and 5 h of the treatment. It also decreased all pro-inflammatory mediators, i.e., NO, TNF-α, and COX-2. It is worth noting that TA increased the activity of enzymes in the liver, e.g., catalase, superoxidase dismutase, and gluthatione peroxidase [37]. In vivo studies conducted by Banno et al. [42] revealed the anti-inflammatory potential of TA in 12-*O*-tetradecanoylphorbol-13-acetate (TPA)-induced inflammation in female ICR mice. Interestingly, TA exhibited not only anti-inflammatory but also antinociceptive activity. The activity was confirmed in an in vivo test of neuropathic pain in mice. The treatment with tormentic acid in a dose of 30 mg/kg p.o. twice a day decreased inflammatory allodynia significantly [92].

### 3.2. Antidiabetic Activity

The antidiabetic and antihyperlipidemic activity of TA was demonstrated in both in vitro and in vivo studies, as presented in Table 2. TA was found to decrease glucose [79,102,103], triglyceride, and fatty acid levels in blood [4,103]. Cell suspension culture of *E. japonica*, where tormentic acid was found as a predominant compound, was administered to high-fat-fed C57BL/6J mice, and the anti-hyperlipidemic and antihyperglycemic effects and mechanism of TA were determined by Shih et al. [4]. The treatment reduced body weight gain and hepatic triacylglycerol content and lowered the weight of white adipose tissue (WAT). Pentacyclic terpenoids from *E. japonica* also increased the content of phosphorylated AMPK-α in the liver and adipose tissue as well. The hypolipidemic effect was reflected in a decrease in the expression of the gene encoding fatty acid synthesis (acyl-coenzyme A: diacylglycerol acyltransferase (DGAT) 2) at doses of 0.5 and 1.0 g/kg of TA. The antidiabetic activity (TA at a 1.0 g/kg dose) was manifested by reduced production of glucose in the liver (through down-regulation of the phosphenolpyruvate carboxyinase (PEPCK) gene), enhanced insulin sensitization, and reduced expression of the 11-β-hydroxysteroid dehydrogenase (11β-HSD1) gene [4]. The study conducted by Wu et al. [103] revealed that TA was effective against induced type 2 diabetes and hyperlipidemia in mice through increased skeletal muscular AMP-activated protein kinase (AMPK) phosphorylation, Akt phosphorylation, and glucose transporter 4 (GLUT4) proteins. The investigations confirmed that TA reduced the hepatic expression of the PEPCK gene and demonstrated down-regulation of the glucose-6-phosphatase (G6 Pase) gene. Furthermore, TA decreased the level of blood triglycerides by decreasing the hepatic sterol regulatory element binding protein 1-c (SREBP-1c) as well as apolipoprotein C-III, and by increasing the expression of peroxisome proliferator activated receptor (PPAR)-α. The other reported mechanism of the antidiabetic activity of TA involved inhibition of protein tyrosine phosphate (PTP1), which enhanced insulin sensitivity of the cells. This indicated promising activity in the treatment of type 2 diabetes [29].

### 3.3. Hepatoprotective Activity

The hepatoprotective properties of TA are related to the anti-inflammatory activity in hepatocytes, e.g., inhibition of the production of pro-inflammatory factors TNF-α, IL-1b, and IL-6 and activation of NF-κB. As reported by Jiang et al. [105], TA was able to decrease the level of thiobarbituric acid reactive substances (TBARS), iNOS, COX-2, TNF-α, IL-1β, and IL-6 and inhibit NF-κB and MAPK activation. Moreover, the antioxidant potential of TA was evaluated by retention of such enzymes as superoxidase dismutase (SOD), glutathione peroxidase (GPx), and catalase (CAT) in the liver. This study indicated that TA might be an interesting agent for prevention of APAP-induced liver injury [105]. TA was also found to be effective against lipopolysaccharide/d-galactosamine-induced fulminant hepatic failure in mice, since a decrease in serum aminotransferase and total bilirubin activities as well as attenuation of histopathological changes were observed [77]. In vivo evaluation of mice demonstrated that dose-dependent treatment (0.75, 1.5, 3 mg/kg) with TA decreased the level of cytochrome C and caspases 3, 8, and 9 and simultaneously increased the expression of the Bcl-2 protein, which protects from apoptosis and thus indicates hepatoprotective activity against injury [77]. Another scientific report revealed that TA exerted a protective effect against liver fibrosis via blocking the PI3k/Akt/mTOR and NF-κB signaling pathways [106]. TA also inhibited the expression of collagen type I and III, which contributed to inhibition of collagen-based extracellular matrix deposition. Treatment with TA reduced aspartate aminotransferase, alanine aminotransferase, and total bilirubin activity significantly [106].

### 3.4. Cardioprotective Activity

The cardioprotective potential of TA was evaluated in vitro in vascular endothelial cells by Shi et al. [107]. TA was found to exert an anti-hypoxic effect by protection of vascular endothelial cells against hypoxia-induced apoptosis via the PI3K/AKT and ERK1/2 signaling pathways. A study conducted by Liu et al. [18] demonstrated that triterpenoids from *A. italica* may be considered as potential cardiomyocyte protective agents. An in vitro model of neonatal rat cardiomyocytes was used for the study. Cells were injured by induced hypoxia and reoxygenation. Treatment with different triterpenoids, including TA, increased the survival rate of the cardiomyocytes significantly. However, the study revealed that the presence of the C-24 aldehyde group in TA may inactivate the cardioprotective effect [18].

### 3.5. Anti-Cancer Activity

As shown in Table 2, TA exhibited anticancer activity against different types of cancer, including leukemia [16], human salivary gland tumor, oral squamous cell carcinoma [38], breast cancer [108], renal cell carcinoma, prostate cancer, melanoma [85], hepatocellular carcinoma [82], cervical cancer [95], adenocarcinoma [29], and colon cancer [58].

The antiproliferative effect on breast cancer was investigated in vitro on the MCF-7 cell line by Zhang et al. [108]. The results revealed that TA increased the generation of intracellular reactive oxygen species (ROS), which led to apoptosis of the cancer cells. It was also noted that the compound activated caspases 3 and 9 and changed the mRNA expression of cyclins, such as cyclin D1 and cyclin-dependent kinase, which led to cell cycle arrest in phase G0/G1. It also decreased the expression of extracellular signal-regulated kinase and inhibited the NF-κB pathway [108]. Scientist evaluated the influence of TA on two-step carcinogenesis initiated by DMBA and then by the TPA promoter in a mouse skin model. As demonstrated by the research, TA reduced the number of papillomas, and the probable mechanism of the antitumor action was related to the kinase C pathway [42]. The activity of TA against hepatocellular carcinoma was visible at a concentration of 15–22.5 µg/mL. The activity involves a dose-dependent increase in the expression of activated CAPS3 and a decrease in poly ADP-ribose polymerase (PARP), which leads to apoptosis and reduces cancer cell viability. It also reduces colony formation as well as cell migration, which can prevent cancer metastasis [82]. In cervical cancer cell lines, as reported by Wu et al. [95], TA inhibits proliferation and induces apoptosis and cell cycle arrest at the G2/M phase. It was also reported to enhance ROS production and block the mTOR/PI3K/AKT pathway [95]. TA was found to exhibit cytotoxic activity to adenocarcinoma cell lines (HeLa) with an IC_50_ value of 33.25 µM [29]. The cytotoxicity of TA against multidrug resistant leukemia was confirmed by Rocha et al. [16]. TA was described to decrease the viability of leukemia cell lines in a dose-dependent manner with an IC_50_ value of 80.25 µM. The mechanism of action was based on the ability to overcome resistance mediated by glycoprotein-P (P-gp) expression [16]. A study conducted by Taniguchi et al. [38] on three cell lines established TA cytotoxicity against human salivary gland tumor (HSG) with an IC_50_ value of 25 µg/mL, human oral squamous cell carcinoma (HSC-2) with an IC_50_ value of 21 µg/mL, and human normal gingival fibroblasts (HGF) with an IC_50_ value of 24 µg/mL. TA also inhibited the early antigen of Epstein-Barr virus (EBV), which is considered a carcinogenesis promoting factor [38]. It was also observed that TA is one of the components responsible for the significant cytotoxic activity of the *n*-hexane and chloroform fractions from *Kleinia pendula* against breast (MCF-7), liver (HepG2), and colon (HCT-116) cancer cell lines [3].

### 3.6. Anti-Osteoarthritic Activity

TA can be used in treatment of osteoarthritis due to its ability to increase the viability of chondrocytes by inhibition of IL-1β-induced apoptosis of these cells in humans [96]. Pretreatment of chondrocytes with different concentrations of TA (2.5, 5, and 10 µM) significantly increased their viability and blocked the apoptosis pathway through inhibition of caspase-3 activity and Bax expression as well as increasing Bcl-2 expression. Moreover, TA was responsible for increased expression of p-PI3K and P-Akt in IL-1β-induced chondrocytes [96].

### 3.7. Antibacterial, Antifungal, Antiviral, and Antiparasitic Activity

The antibacterial and antifungal activity of tormentic acid was confirmed during in vitro and in vivo studies. TA was evaluated as a potent agent against *Pseudomonas aeruginosa* [91], *Staphylococcus aureus* [28,66], and *Candida albicans* [28]. It also exhibited antiviral activity in vitro against HIV virus. The compound inhibited HIV-1 protease at a concentration of 17.9 µg/mL [71]. In vivo evaluation by Ghosh et al. [91] demonstrated the antibacterial activity of TA from *Sarcochlamys pulcherrima* against *P. aureginosa* in a dose of 55 µg/mL, which had the highest diameter of the zone of inhibition. The mechanism of such activity involved increased cytoplasmic membrane depolarization leading to disintegration of the bacterial cell. Moreover, the anti-biofilm effect of TA against *P. aeruginosa* biofilm was revealed and effective doses of TA were found to be safe for mice. The inhibition of biofilm formation was shown to be mediated via suppressed secretion of pyoverdin, protease, and swarming motility of *P. aeruginosa* [91]. *Staphylococcus aureus* is a gram-positive bacteria regarded as the main nasopharyngeal inflammation factor. As shown in a study conducted by Mashezha et al. [66], TA in a 100 µg/mL dose partially inhibited the growth of *S. aureus* (72.7%), while the production of extracellular proteases by the bacteria was inhibited completely. Therefore, TA should be further investigated as a potential agent for antivirulence therapy to combat *S. aureus* infections. The study reported by Dimitrova et al. [28] also revealed that TA was effective against *S. aureus* probably due to adsorption of polyphenols (including TA) to the bacterial membranes leading to their disruption and leakage of cellular content and generation of hydroperoxides from polyphenols. The authors also demonstrated the antifungal activity of TA against *Candida albicans* in an in vitro study [28]. 

The antiparasitic activity of fractions obtained from *Pourouma guianensis* (Moraceae) leaves containing several terpenoids, including tormentic acid, was evaluated by Torres-Santos et al. [90]. They found that triterpenoids from *P. guianensis* were useful for the development of new antileishmanial drugs, since leishmaniasis is an insect-borne protozoan infection affecting millions of people worldwide.

### 3.8. Neuroprotective Activity

TA was examined for its neuroprotective activity as a potent agent in the treatment of Alzheimer’s disease (AD), memory impairment [94], Parkinson’s disease, and other inflammations in the nervous system [109]. In in vivo test on transgenic mice with AD, tormentic acid exerted a neuroprotective effect against neuro-inflammation and memory impairment [94]. Treatment with 5 or 10 mg/kg of TA decreased the number of GFAP-positive astrocytes and CD11b-positive microglia in the pre-frontal cortex (PFC) and hippocampus (HC). The secretion of pro-inflammatory factors (e.g., TNF-α, IL-1β, IL-6) in the AD mice was decreased after the treatment with TA. Additionally, TA reduced the area taken by the Aβ plaque in PFC and HC. To evaluate the degree of neuron survival, the scientists evaluated the anti-apoptotic activity of TA. It turned out that TA was able to decrease DNA fragmentation and levels of caspase-3 activity, resulting in improved neuron survival. Moreover, pretreatment with TA suppressed the nuclear translocation of NF-κB p65 induced by Aβ exposure in BV2 microglial cells [94].

## 4. Derivatives of Tormentic Acid

**Figure 1:**
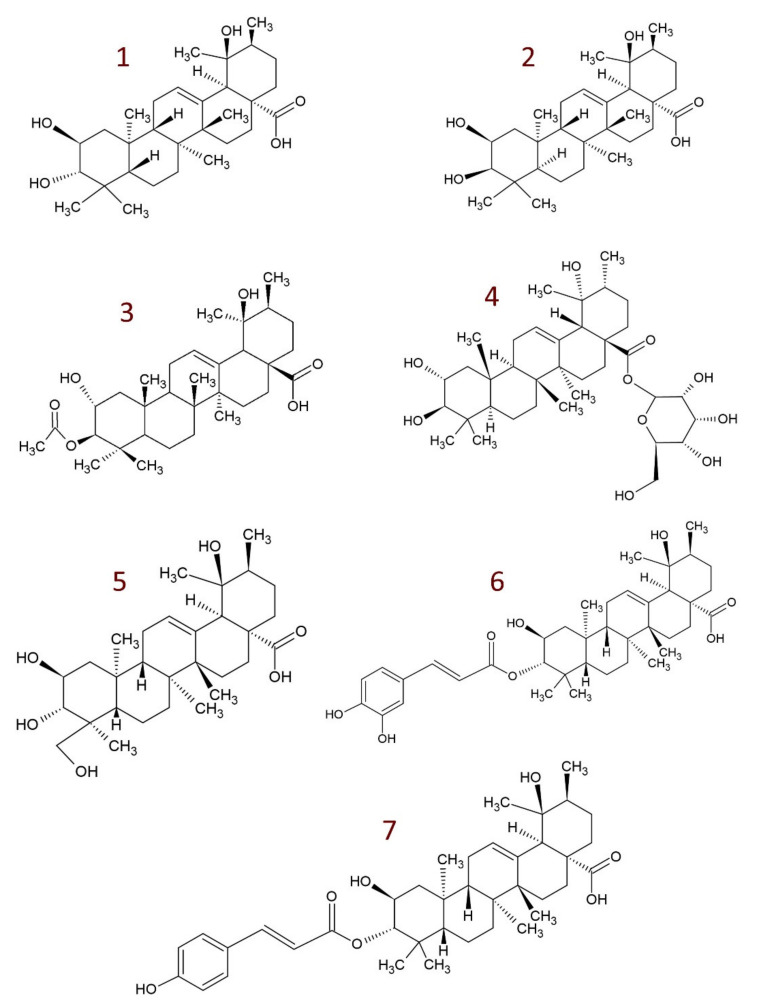
Structure of tormentic acid (**1**) and its common natural derivatives; **2**—euscaphic acid; **3**—3β-acetyl tormentic acid; **4**—rosamultin; **5**—23-hydroxytormentic acid; **6**—3-*O*-*trans*-caffeoyltormentic acid; **7**—3-*O*-*cis*-*p*-coumaroyltormentic acid.

Although tormentic acid (TA) is found in a variety of plants in its “basic form”, it also occurs in the form of various derivatives. Some common structures are shown in Figure 1. TA and its derivatives are found in commonly known cultivated and consumed fruits or vegetables, e.g., strawberries [110], rose fruits [81], apples [73], and quince [34].

The reported TA derivatives include:

- euscaphic acid (EA)—a stereoisomer of tormentic acid [9,27,32,50,111,112,113];
- 2-epi-tormentic acid (2β,3β,19α-trihydroxy-urs-12-en-28-oic acid) [9,114];
- acetylated compounds, e.g., 3β-acetyl tormentic acid; 2α-acetyl tormentic acid [16,115,116,117]
- hydroxylated derivatives, e.g., 23-hydroxytormentic acid [27]; 24-hydroxytormentic acid [64,118]; 11α-hydroxytormentic acid [53,110,112]; hydroxytormentic acid [53];
- coumaroyl esters, e.g., 3-*O*-*cis*-*p*-coumaroyltormentic acid; 3-*O*-*trans*-*p*-coumaroyltormentic acid [6,22,119];
- caffeoyl esters, e.g., 3-*O*-*trans*-caffeoyltormentic acid [6,69];
- glucosides, e.g., tormentic acid 3β-*O*-β-d-quinovopyranoside; tormentic acid 3β-*O*-β-d-fucopyranoside; tormentic acid 3β-*O*-β-d-rhamnopyranoside; rosamultin (tormentic acid 28-*O*-glucoside) [27,32,110,120,121]; tormentic acid β-d-glucopyranosyl ester [74,122];
- others, e.g., 6-methoxy-β-glucopyranosyl ester [112]; dihydrotormentic acid and methoxytormentic acid [110]; 3b-*p*-hydroxybenzoyloxytormentic acid [123]; (3*R*,19*R*)-methyl-3,19-dihydroxy-2-oxo-urs-12-en-28-carboxylate; (2*R*,19*R*)–methyl-2,19-dihydroxy-3-oxo-urs-12-en-28-carboxylate; (19*R*)-methyl-2,19-dihydroxyursa-3-oxo-1,12-dien-28-carboxylate; (2*S*,3*R*,19*R*)–methyl-2,3,19-trihydroxyurs-12-en-28-carboxylate; (2*R*,3*R*,19*R*)-2,3-bis(acetyloxy)-19-hydroxyurs-12-en-28-carboxylic acid; (2*R*,3*R*,19*R*)-2-acetyloxy-3,19-dihydroxyurs-12-en-28-carboxylic acid; (2*R*,3*R*,19*R*)-3-acetyloxy-2,19-dihydroxyurs-12-en-28-carboxylic acid; (3*R*,19*R*)–methyl-3-acetyloxy-19-hydroxy-2-oxo-urs-12-en-28-carboxylate; (2*R*,19*R*)-methyl-2-acetyloxy-19-hydroxy-3-oxo-urs-12-en-28-carboxylate; (2*R*,3*R*,19*R*)–methyl-2,3-bis(chloroacetyloxy)-19-hydroxy-urs-12-en-28-carboxylate; (2*R*,3*R*,19*R*)–methyl-2-chloroacetyloxy-3,19-dihydroxyurs-12-en-28-carboxylate; (2*R*,3*R*,19*R*)–methyl-3-chloroacetyloxy-2,19-dihydroxyurs-12-en-28-carboxylate [9].

TA derivatives can be found in many species of several plant families (e.g., Combretaceae, Lamiaceae, Utricaceae, Lauraceae Oleaceae, and Rosaceae). Many of these compounds possess confirmed biological activity. Several TA glycosides have been described: tormentic acid 3β-*O*-β-d-quinovopyranoside, tormentic acid 3β-*O*-β-d-fucopyranoside, tormentic acid 3β-*O*-β-d-rhamnopyranoside, etc. [14,32,74,120].

One of the best-known derivatives is 3β-acetyl tormentic acid. The compound was found to be an inhibitor of the ABCC subfamily of transporters (with selectivity for MRP1/ABCC1). It may be potentially used as a co-adjuvant in the treatment of multidrug resistant tumors [116]. Another derivative, 24-hydroxytormentic acid, which can be found, e.g., in *Triumfetta cordifolia* (Tiliaceae) [64] or *Ocotea* *suaveolens* (Lauraceae), was reported by Beirith et al. [118] to be an antinociceptive agent; however, its mechanism of action has not been revealed. 

Two coumaroyl derivatives have been identified in the callus culture of *E. japonica* leaves, i.e., 3-*O*-*cis*-*p*-coumaroyltormentic acid and its isomer 3-*O*-*trans*-*p*-coumaroyltormentic acid [38]. Interestingly, both structures have not been reported in the leaves of the source plant. The coumaroyl derivatives exerted an anti-tumor effect on human oral cell lines. Furthermore, 3-*O*-(*E*)-*p*-coumaroyl tormentic acid was found to induce caspase-dependent apoptotic cell death in human leukemia cell lines due to its cytotoxic activity and inhibition of Topo I, which was confirmed by Kikuchi et al. [119]. In addition, 3-*O-trans*-caffeoyltormentic acid was detected in *Eriobotrya deflexa* f. *buisanensis* leaves (Rosaceae), where it was shown to possess anti-dengue virus activity [69]. 

One of the most important TA derivatives is euscaphic acid (EA), which is actually a stereoisomer of tormentic acid. Both TA and EA are sometimes detected or isolated in parallel [27,83]. EA was reported to occur in leaves of many species, e.g., *Rosa laevigata* Michx. and *Rubus crataegifolius* Bunge, and in roots of *Rosa rugosa* Thunb. and *Myrianthus arboreus* P. Beauv. [49,111,112]. It exhibits anti-inflammatory activity by decreasing the production of prostaglandin E_2_, nitric oxide, tumor necrosis factor-alpha, cyclooxygenase-2, and interleukin 1-β. It also decreases the transcriptional activity of nuclear factor Kappa B (NF-κB), as described by Kim et al. [111]. EA has been found to play a significant role in suppressing the proliferation of nasopharyngeal carcinoma cell lines. The compound induced cell apoptosis and cell cycle arrest in the G1/S phase by inhibition of the expression of phosphatidylinositide 3-kinases, phosphorylated protein kinase B, and phosphorylated mammalian target of rapamycin in carcinoma cell lines [113].

Two tormentic acid *O*-hexosides and several methyl or hydroxyl derivatives of tormentic and dihydrotormentic acids have been recently found in strawberries: methoxytormentic acid (or hydroxytormentic acid methylester), methoxydihydrotormentic acid *O*-hexoside (or *O*-hexoside of hydroxytormentic acid methylester), and methoxytormentic acid *O*-hexoside (or *O*-hexoside of hydroxytormentic acid methylester) [110]. Besides, 3β-*p*-hydroxybenzoyloxytormentic acid has been found in *Luehea divaricate* Mart. (Tiliaceae) [123].

Moreover, the study conducted by Csuk et al. [9] investigated several derivatives of tormentic acid for their antitumor activity. Among them, (2*R*,3*R*,19*R*)methyl 2,3-bis(chloroacetyloxy)-19-hydroxyurs-12-en-28-carboxylate was found to be a very potent antitumor agent acting through an apoptosis-induction pathway. The cytotoxicity of this compound was tested in a panel of various cancer cell lines, e.g., 518A2 (melanoma), 8505C (anaplastic thyroid), A253 (head), A2780 (ovarian), A549 (lung), DLD1 (colon), and MCF7 (mamma) [9].

## 5. Conclusions

TA has confirmed pleiotropic activity and can significantly affect different biochemical mechanisms involved in development of various medical conditions. To date, many botanical sources of TA have been discovered, including edible and medicinal plants. The large number of plant families and species may suggest that further natural sources of TA may still be waiting to be discovered. Challenges related to the effective medicinal and industrial application of TA may include application of large-scale biotechnological methods, optimization of extraction conditions/techniques, and development of the best administration routes. It is also reasonable to continue to seek, discover, and investigate natural or synthetic TA derivatives.

## Figures and Tables

**Table 1:** Confirmed botanical sources of tormentic acid (currently accepted botanical names (where applicable) according to www.theplantlist.org (accessed on 12 April 2021) are given in square brackets) and initial extrahent type used for elution of TA from plant material.

Plant Family	Species and Organ Investigated	Extraction Solvent	Ref.
Acanthaceae	*Rostellularia procumbens* (L.) Nees [*Justicia procumbens* L.] Whole plant	80% Ethanol	[51]
Aphloiaceae	*Aphloia theiformis* (Vahl) Benn. Leaves	Methanol	[52]
Aphloiaceae	*Aphloia theiformis* (Vahl) Benn. Leaves	70% Ethanol	[53]
Betulaceae	*Betula schmidtii* Regel Twigs	80% Methanol	[15]
Bignoniaceae	*Markhamia obtusifolia* (Baker) Sprague Leaves	Acetone	[54]
Bignoniaceae	*Markhamia platycalyx* (Baker) Sprague [*Markhamia lutea* (Benth.) K.Schum.] Leaves	95% Ethanol	[55]
Bignoniaceae	*Markhamia tomentosa* (Benth) K. Schum ex Engl. Leaves	Ethanol	[19]
Boraginaceae	*Anchusa italica* Retz. [*Anchusa azurea* Mill.] Aerial parts	75% Ethanol	[18]
Boraginaceae	*Arnebia euchroma* (Royle) I.M.Johnst. Roots	Methanol	[56]
Caprifoliaceae	*Cephalaria tuteliana* Kuș & Göktürk Not specified	Methanol	[20]
Caryophyllaceae	*Psammosilene tunicoides* W.C. Wu & C. Y. Wu. Roots	80% Ethanol	[57]
Compositae	*Kleinia pendula* (Forssk.) DC. Fresh aerial parts	Methanol	[3]
Ericaceae	*Rhododendron websterianum* Rehder & E.H. Wilson Fruits	95% Ethanol	[21]
Lamiaceae	*Hyptis capitata* Jacq. Leaves and stems	Methanol	[58]
Lamiaceae	*Isodon rubescens* (Hemsl.) H.Hara Whole plant	-	[59]
Lamiaceae	*Lavandula luisieri* (Rozeira) Riv.-Mart. [*Lavandula stoechas* subsp. *luisieri* (Rozeira) Rozeira] Flowering plant	Ethanol	[41]
Lamiaceae	*Leptohyptis macrostachys* (L’H’erit.), Harley and J.F.B. Pastore (previously *Hyptis macrostachys* Benth.) Aerial parts	95% Ethanol	[60]
Lamiaceae	*Ocimum gratissimum* L. Aerial parts	Methanol	[61]
Lamiaceae	*Perilla frutescens* L. Britton Cell culture from leaves	Methanol	[45]
Lamiaceae	*Perilla frutescens* (L.) Britton var. acuta Kudo Fresh leaves	Methanol	[47]
Lamiaceae	*Perilla frutescens* (L.) Britton Leaves	Ethanol	[42,46]
Lamiaceae	*Platostoma rotundifolium* (Briq.) A. J. Paton Aerial parts	Ethyl acetate	[62]
Lamiaceae	*Salvia judaica* Boiss. Aerial parts	Ethanol	[43]
Lamiaceae	*Salvia miltiorrhiza* Bunge Roots and aerial parts	Ethanol	[44]
Leguminosae	*Campylotropis hirtella* (Franch.) Schindl. Roots	-	[63]
Malvaceae	*Triumfetta cordifolia* A.Rich. Stems	Methylene: methanol (1:1)	[64]
Myrtaceae	*Acca sellowiana* (O.Berg) Burret Callus culture from fruit pulp	Methanol	[65]
Myrtaceae	*Callistemon citrinus* (Curtis) Skeels Leaves	Dichloromethane: Methanol (50:50, v/v) Water: Ethanol (50:50, v/v)	[66]
Oleaceae	*Ligustrum robustum* (Roxb.) Blume Not specified	70% Methanol	[22]
Oleaceae	Olea europaea L. Cell-suspension cultures (callus induced from leaf stalk)	Methanol	[23]
Oleaceae	*Olea europaea* L. (varieties Manzanilo, Picual, Koroneiki, and Coratina) Fruits	Methanol	[67]
Oleaceae	*Osmanthus fragrans* Lour Fruits	Methanol	[7]
Polygonaceae	*Rumex japonicus* Houtt. Stems	80% Ethanol	[24]
Rosaceae	*Agrimonia pilosa* Ledeb. Aerial parts	80% Ethanol	[29]
Rosaceae	*Alchemilla faeroensis* (J. Lange) Buser Aerial parts	Ethanol	[26]
Rosaceae	*Cotoneaster simonsii* hort. ex Baker Aerial parts (leaves and twigs)	Chloroform	[68]
Rosaceae	*Crataegus pinnatifida* Bunge Leaves	80% Ethanol	[30]
Rosaceae	*Cydonia oblonga* Mill. Seeds	Methanol	[34]
Rosaceae	*Eriobotrya deflexa* f. *buisanesis* [*Eriobotrya deflexa* (Hemsl.) Nakai.] Leaves	Methanol	[69]
Rosaceae	*Eriobotrya fragrans* Champ. ex Benth Leaves	95% Ethanol	[70]
Rosaceae	*Eriobotrya japonica* (Thunb) Lindl. Leaves	80% Methanol	[36]
Rosaceae	*Eriobotrya japonica* (Thunb.) Lindl. Leaves	95% Ethanol	[31,35]
Rosacae	*Eriobotrya japonica* (Thunb.) Lindl Cell suspension culture (callus induced from leaves)	Ethanol	[37]
Rosaceae	*Eriobotrya japonica* (Thunb.) Lindl. Callus cultures induced from an axenic leaf	Ethanol	[38]
Rosaceae	*Eriobotrya japonica* (Thunb) Lindl. Cell suspension culture (obtained from immature embryos)	95% Ethanol	[39]
Rosaceae	*Eriobotrya japonica* (Thunb.) Lindl. Cell suspension culture (callus induced from leaves)	95% Ethanol	[4]
Rosaceae	*Fragaria × ananassa* Duch. var ‘Falandi’ Fresh fruit	95% Ethanol	[33]
Rosaceae	*Fragaria × ananassa* Duch. var ‘Hokouwase’ Green unripe fresh fruit	Methanol	[14]
Rosaceae	*Geum japonicum* auct. [*Geum macrophyllum* Willd.] Whole plant	Methanol	[71]
Rosaceae	*Geum rivale* L. Flowering aerial parts	Chloroform: Methanol (9:1)	[72]
Rosaceae	*Geum urbanum* L. Roots and aerial parts	Methanol	[28]
Rosaceae	*Malus domestica* Borkh varieties “Mela Rosa Marchigiana” and “Golden Delicious” Pulp callus culture	Methanol	[73]
Rosaceae	*Margyricarpus setosus* Ruiz & Pav. [*Margyricarpus pinnatus* (Lam.) Kuntze] Aerial parts	Methanol	[74]
Rosaceae	*Potentilla anserina* L. Roots	-	[75]
Rosaceae	*Potentilla anserina* L. Roots	70% Ethanol	[76]
Rosaceae	*Potentilla chinensis* Ser. Whole plant	95% Ethanol	[77]
Rosaceae	*Potentilla fulgens* [*Potentilla lineata* Trevir.] Roots	Methanol	[78]
Rosaceae	*Poterium ancistroides* Desf. [*Sanguisorba ancistroides* (Desf.) Ces.] Aerial parts	Ethyl acetate	[79]
Rosaceae	*Poterium ancistroides* Desf. [*Sanguisorba ancistroides* (Desf.) Ces.] Herb	Methanol	[80]
Rosaceae	*Rosa nutkana* C.Presl Fruits	Methanol	[81]
Rosaceae	*Rosa roxburghii*	-	[82]
Rosaceae	*Rosa rugosa* Thunb. Roots	Methanol	[32]
Rosaceae	*Rubus chingii* Hu Roots and rhizomes	Ethanol	[83]
Rosaceae	*Rubus crataegifolius* Bunge Leaves	Methanol	[27]
Rosaceae	*Sanguisorba officinalis* L. Root	Cold water Hot water Methanol	[84]
Rosaceae	*Sarcopoterium spinosum* (L.) Spach. Aerial parts	-	[85]
Rubiaceae	*Knoxia valerianoides* Thorel ex Pit. [*Knoxia roxburghii* subsp. brunonis (Wall. ex G.Don) R.Bhattacharjee & Deb] Roots	Ethanol	[86]
Sapotaceae	*Tridesmostemon omphalocarpoides* Engl. Wood and stem bark	Dichloromethane: Methanol (1:1)	[87]
Saxifragaceae	*Tiarella polyphylla* D. Don Whole plant	Methanol	[17]
Staphyleaceae	*Euscaphis konishii* Hayata [*Euscaphis japonica* (Thunb.) Kanitz] Twigs	95% Ethanol	[88]
Urticaceae	*Cecropialyratiloba* Miq. [*Cecropia pachystachya* Trécul.)] Roots	Methanol	[16]
Urticaceae	*Cecropia pachystachya* Trécul Roots, root bark, stem and stem bark	Ethanol	[25]
Urticaceae	*Debregeasia salicifolia* D. Don. [*Debregeasia saeneb* (Forssk.) Hepper & J.R.I.Wood] Stems	Methanol	[5]
Urticaceae	*Myrianthus arboreus* P.Beauv Stem bark	Methylated ethyl acetate	[50]
Urticaceae	*Myrianthus arboreus* P.Beauv Root wood	Methylated spirit	[48]
Urticaceae	*Myrianthus arboreus* P.Beauv Stems	Chloroform	[49]
Urticaceae	*Myrianthus serratus* (Trecul) Benth. Trunk wood	Ethyl acetate	[89]
Urticaceae	*Pourouma guianensis* Aubl. Leaves	Methanol	[90]
Urticaceae	*Sarcochlamys pulcherrima* (Roxb.) Gaudich. Aerial parts	Methanol	[91]
Vochysiaceae	*Vochysia divergens* Pohl. Stem bark	Ethanol	[92,93]

**Table 2:** Pharmacological activity of tormentic acid.

Biological Activity	Model	Ref.
Anti-inflammatory (anti-osteoarthritic): –decreasing the interleukin (IL)-1β-stimulated expression of MMP-3 and MMP-13; –inhibition of the IL-1β-induced expression of iNOS and COX-2, and the production of PGE2 and NO; inhibition of IL-1β-induced NF-κB activation	In vitro Human Articular Chondrocyte Culture	[97]
Anti-inflammatory: –inhibition of nitric oxide (NO) and prostaglandin E 2 (PGE 2) production by inhibiting iNOS and COX-2 expression; –inhibition of LPS-stimulated production of TNF-α and IL-1β; –activation of LXRα (liver X receptor α) and inhibition of LPS-induced NF-κB activation	In vitro BV2 microglial cells	[98]
Antioxidative and anti-inflammatory: –decreasing reactive oxygen species (ROS) generation; –inhibition of the expression of inducible nitric oxide synthase (iNOS) and NADPH oxidase (NOX); –decreasing the production of tumor necrosis factor-α (TNF-α), interleukin 6 (IL-6), and IL-1β; –preventing phosphorylation of nuclear factor-κB (NF-κB) subunit p65 and degradation of NF-κB inhibitor α (IκBα)	In vitro Rat vascular smooth muscle cells (RVSMCs);	[99]
Anti-inflammatory: –decreasing paw edema; –increasing the activities of catalase (CAT), superoxide dismutase (SOD), and glutathione peroxidase (GPx) in liver tissue; –attenuating the level of thiobarbituric acid reactive substances (TBARS) in the edematous paw; –decreasing the nitric oxide (NO) levels at the serum level and diminishing the serum tumor necrosis factor (TNF-α); –decreasing the expression of inducible nitric oxide synthase (iNOS) and cyclooxygenase-2 (COX-2)	Ex vivo and in vivo RAW264.7 macrophages and λ-carrageenin-induced hind paw edema model in mice	[37]
Anti-inflammatory: –reducing the production of NO, prostaglandin E2 (PGE2), and tumor necrosis factor-α (TNF-α) induced by LPS; –suppressing the LPS-induced expression of inducible nitric oxide synthase (iNOS), cyclooxygenase-2 (COX-2), and TNF-α at the mRNA and protein levels; –decreasing DNA binding of nuclear factor kappa B(NF-kB) and nuclear translocation of the p65 and p50 subunits of NF-kB; –suppressing degradation and phosphorylation of inhibitor of kappa B-Alpha	In vitro LPS stimulated RAW264.7 cells	[100]
Anti-inflammatory/antinociceptive (20–30 mg/kg)	In vivo Writhing Assay; Hot-Plate Test; Carrageenan-Induced Edema in Sprague–Dawley Rats	[32]
Anti-inflammatory: –inhibition of the production of interleukin-6 and interleukin-8; –inhibition of TLR4 (Toll-like receptor 4) expression; –inhibition of activation of nuclear factor kappa B (NF-κB); –inhibition of activation of mitogen-activated protein kinases (MAPKs)	In vitro LPS-stimulated human gingival fibroblasts (HGFs)	[101]
Anti-inflammatory: –inhibition of LPS-induced NO production	In vitro	[69]
Anti-inflammatory: –inhibitory effect on IFN-γ secretion –inhibition of COX-1 and COX-2 –apoptosis-inducing effect	In vitro LPS-stimulated Raw 264.7 macrophage	[61]
–Anti-inflammatory; –Potent inhibitory effect on Epstein-Barr virus early antigen (EBV-EA) activation; –Antitumor-promoting activity (strong)	In vivo*:* –TPA-induced ear edema inflammation in mice; –two-stage carcinogenesis test of mouse tumor; In vitro EBV-EA activation experiment	[42]
–Cytotoxic activity against the HeLa cell line; –Antidiabetic activity –Inhibition of PTP1B (Protein tyrosine phosphate)	In vitro	[29]
Cytotoxic to sensitive and multidrug resistant leukemia cell lines; Active toward a multidrug resistant (MDR) leukemia cell line overexpressing glycoprotein-P (P-gp)	In vitro (anti-MDR activity in Lucena-1, a leukemia cell line that overexpresses P-gp and presents cross resistance to several unrelated cytotoxic drugs)	[16]
Cytotoxic	In vitro HCT-8, A549, P-388, L-1210 tumor cell lines	[58]
–Cytotoxicity in human oral tumor cell lines: human salivary gland tumor and human oral squamous cell carcinoma –Inhibition of the activation of Epstein–Barr virus early antigen (EBV-EA)	In vivo EBV genome-carrying lymphoblastoid cells In vitro human oral squamous cell carcinoma (HSC-2), human salivary gland tumor (HSG)	[38]
Antidiabetic and antihyperlipidemic: –Antihyperlipidemic: decreasing gene expressions of fatty acids, increasing the content of phosphorylated AMPK-α in liver and adipose tissue, inhibition of DGAT 1 expression, and decreasing the level of triglycerides in blood –Antidiabetic: down-regulation of phosphenolpyruvate carboxykinase (PEPCK), improving insulin sensitization (at 1.0 g/kg), and decreasing the expression of the hepatic and adipose 11-β-hydroxysteroid dehydroxygenase (11β-HSD1) gene	In vivo high-fat fed C57BL/6J mice	[4]
Hypoglycemic: decreasing the blood glucose level (at 10 mg/kg)	In vivo normoglycemic Wistar rats	[79]
Hypoglycemic effect (at 30 mg/kg): –decreasing glucose levels in normal rats; –increasing fasting plasma insulin levels Acute toxicity not observed (at 600 mg/kg, intraperitoneally)	In vivo normoglycemic, hyperglycemic, and streptozotocin-induced diabetic Wistar rats	[80]
Hypoglycemic effect: –direct stimulation of insulin secretion by pancreatic islets of Langerhans	In vitro pancreatic islets of Langerhans isolated from fed Wistar rats	[102]
Antidiabetic: –inhibition of alfa-glucosidase	In vitro	[78]
Antidiabetic and antihyperlipidemic activity: –lowering blood glucose, triglycerides, free fatty acids, leptin levels; –decreasing the area of adipocytes and ballooning degeneration of hepatocytes; –reducing visceral fat mass, reducing hepatic triacylglycerol contents; –enhancing skeletal muscular Akt phosphorylation and increasing insulin sensitivity; –decreasing blood triglycerides by down-regulation of the hepatic sterol regulatory element binding protein-1c (SREBP-1c) and apolipoprotein C-III (apo C-III) and increasing the expression of peroxisome proliferator activated receptor (PPAR)-α	In vivo C57BL/6J mice with induced type 2 diabetes and hyperlipidemia	[103]
Influencing the processes present in vasculoproliferative diseases (diseases related to vascular smooth muscle cell (VSMC) abnormal proliferation): –increasing apoptosis of serum-deprived A7r5 cells and inhibiting A7r5 cell proliferation; –rapid induction of significant modifications in the vascular smooth muscle cell (VSMC) phenotype; –inhibition of VSMC proliferation and VSMC cell death	In vitro Clonal rat embryonic VSMCs (A7r5) and human umbilical vein endothelial cells (HUVEC)	[93]
Anti-melanogenesis effect (melanin synthesis inhibitory activity with less cytotoxicity) Antibacterial activity against *Propionibacterium acnes* Promotion of skin collagen synthesis	In vitro Mouse melanoma cell line B16; *Propionibacterium acnes* (NBRC 107605)	[104]
Hepatoprotective (preventing fulminant hepatic failure): –blocking the NF-κB signaling pathway for anti-inflammatory response (alleviating the pro-inflammatory cytokines, e.g., TNF-α and NO/iNOS by inhibiting nuclear factor-κB activity); –inhibition of hepatic lipid peroxidation; –decreasing serum aminotransferase and total bilirubin activities; –attenuating hepatocellular apoptosis	In vivo lipopolysaccharide/d-galactosamine-induced acute hepatic failure in male C57BL/6 mice	[77]
Hepatoprotective: –inhibition of the production of pro-inflammatory factors such as: tumor necrosis factor-alpha (TNF-α), interleukin-1beta (IL-1β), and IL-6; –inhibition of inducible NO synthetase (iNOS) and cyclooxygenase-2 (COX-2); –inhibition of nuclear factor –κB (NF-κB) activation; –inhibition of the activation of mitogen-activated protein kinases (MAPKs); –retention of enzymes (essential for the antioxidative properties of liver): superoxidase dismutase (SOD), glutathione peroxidase (GPx), catalase (CAT)	In vivo Acetaminophen-induced hepatotoxicity in male ICR mice	[105]
Protective effect against liver fibrosis: –inhibition of the activation of hepatic stellate cells; –reducing aspartate aminotransferase, alanine aminotransferase, and total bilirubin activity; –inhibition of expression of collagen type I and III; alleviation of collagen-based extracellular matrix deposition; –promoting cell apoptosis via blocking of the PI3K/Akt/mTOR and NF-κB signaling pathways	In vitro Hepatic stellate cells (HSCs) stimulated with platelet-derived growth factor-BB	[106]
Cardioprotective (protective effects on hypoxia/reoxygenation (H/R)-induced cardiomyocyte injury)	In vitro Neonatal rat cardiomyocytes subjected to hypoxia/reoxygenation (H/R) insult	[18]
Anti-hypoxic (protecting vascular endothelial cells against hypoxia-induced damage via the PI3K/AKT and ERK 1/2 signaling pathway)	In vitro (EA.hy926 cells)	[107]
Antiproliferative: –causing apoptosis and G0/G1 phase arrest in cancer cell lines; –induction of cell cycle arrest via changing the cyclin D1 and cyclin-dependent kinase 4 mRNA expression levels; –down-regulation of the NF-kappa-B cell survival pathway and the expression level of phosphorylated ERK (extracellular signal-regulated kinase)	In vitro Cancer cell lines: human hepatoma cells HepG-2 and Bel-7402, lung cancer cell A549, breast cancer cell MCF-7 Normal LO2 cell line	[108]
Antiproliferative	In vitro	[85]
Anti-cancer (anti-hepatocellular carcinoma activity): –decreasing cell viability, colony formation, and cell migration; –induction of apoptosis; –changing the levels of caspase-3 and poly ADP-ribose polymerase expression	In vitro Hepatocellular carcinoma cells (HepG2, Bel-7405, Sk-hep-1)	[39]
Anti-cancer: –induction of cell cycle arrest; –enhancement of ROS production; –targeting the mTOR/PI3K/AKT signaling pathway in cisplatin-resistant human cervical cancer cells	In vitro Cisplatin-resistant human cervical cancer cells (HeLa cells)	[95]
Anti-osteoarthritic (inhibition of IL-1β-induced chondrocyte apoptosis by activation of the PI3K/Akt signaling pathway): –inhibition of IL-1β induced cytotoxicity and apoptosis in chondrocytes; –increasing B-cell lymphoma (Bcl)-2 expression; –decreasing capsase-3 activity and Bax expression; –increasing the expression of p-PI3K and p-Akt in IL-1β-induced chondrocytes	In vitro IL-1β-treated human osteoarthritic chondrocytes	[96]
Antinociceptive (anti-allodynic)	In vivo two models of chronic pain (neuropathic pain and inflammatory pain) in mice	[92]
Antibacterial	In vitro	[51]
Antibacterial and antibiofilm effect: –inhibition of growth of *P. aeruginosa*; –depolarization of bacterial *P. aeruginosa* membrane; –inhibition of biofilm formation due to suppressed secretion of pyoverdine and suppressed secretion of protease and swarming motility of *P. aeruginosa*	In vivo Mouse model of catheter infection for evaluation of antibiofilm activity and BALB/c mouse model for determination of in vivo toxicity In vitro *P. aeruginosa* cultures; murine macrophage cell line (RAW 264.7) for cytotoxicity assay	[91]
Antibacterial against *S. aureus* Antifungal against *C. albicans*	In vitro	[28]
Antibacterial against *S. aureus*	In vitro	[81]
Bacteriostatic against *S. aureus*: –inhibition of extracellular protease production resulting in inhibition of *S. aureus* growth	In vitro	[66]
Antivirus: inhibition of virus HIV-1 protease	In vitro	[71]
Insect antifeedant	In vivo *Spodoptera littoralis* L6 larvae	[41]
Neuroprotective: –protecting against neurotoxicity (preventing neuronal loss); –blocking MPP^+^-induced apoptosis; –inhibiting intracellular accumulation of reactive oxygen species (ROS); –protecting from neuronal death through reversing the inhibition of the PI3-K/Akt/GSK3b pathway	In vitro Parkinson’s disease cellular model: MPP^+^-induced neurotoxicity in human neuroblastoma SH-SY5Y cells	[109]
Neuroprotective: –decreasing amyloid plaque deposition; –reducing microglial activation and decreasing the secretion of pro-inflammatory factors; –suppressing the production of pro-inflammatory markers and the nuclear translocation of nuclear factor-κB (NF-κB); –reducing inhibited neurotoxicity and improving neuron survival	In vivo Amyloid β precursor protein (APP)/presenilin 1 (PS1) transgenic mice In vitro BV2 microglia cells	[94]
